# Inhibition of Transforming Growth Factor-β Receptor signaling promotes culture expansion of undifferentiated human Endometrial Mesenchymal Stem/stromal Cells

**DOI:** 10.1038/srep15042

**Published:** 2015-10-13

**Authors:** Shanti Gurung, Jerome A. Werkmeister, Caroline E. Gargett

**Affiliations:** 1 Department of Obstetrics and Gynaecology, Monash University, Victoria, 3800 Australia; 2 Hudson Institute of Medical Research, 27-31 Wright Street, Clayton, Victoria, 3168 Australia; 3 CSIRO Manufacturing Flagship, Bag 10, Clayton South, Victoria, 3168 Australia

## Abstract

Human endometrial MSC (eMSC) are a novel source of MSC easily harvested from the highly regenerative uterine lining. We have developed protocols for eMSC isolation from single cell suspensions using magnetic bead-sorting using a perivascular marker antibody to SUSD2 and culture expansion in serum free medium (SFM). Similar to other MSC, eMSC spontaneously differentiate into fibroblasts during culture expansion decreasing their purity and efficacy. The aim of this study was to determine if A83-01, a TGF-β receptor inhibitor prevents eMSC differentiation in culture. SUSD2^+^ eMSC were cultured in SFM with bFGF/EGF in 5% O_2_/5% CO_2_. At passage 6, eMSC were incubated with or without A83-01 for 7 days, then analysed for MSC properties. A83-01 dose dependently promoted SUSD2^+^ eMSC proliferation and blocked apoptosis via the SMAD 2/3 pathway. Fewer A83-01 treated cells were autofluorescent or stained with β-galactosidase, indicating reduced senescence. A83-01-treated cells had higher cloning efficiency, differentiated into mesodermal lineages and expressed MSC phenotypic markers. These data suggest that A83-01 maintains SUSD2^+^ eMSC stemness, promoting proliferation by blocking senescence and apoptosis in late passage cultures through binding to TGF-β receptors. Small molecules such as A83-01 may enable the expansion of undifferentiated MSC for use in tissue engineering and cell-based therapies.

Mesenchymal stem/stromal cells (MSC) have been identified in almost all adult human tissues^1^ since Friedenstein and colleagues discovered colony-forming fibroblasts in bone marrow in the 1970s^2^. MSC are typically characterised by their clonogenicity, multipotency^3^ and surface phenotype^4^. In addition, MSC home to damaged tissues^5^, and have anti-inflammatory and immunomodulatory properties^6^. Increasingly, MSC are recognized for their biological effects in repairing damaged tissues through secretion of soluble bioactive molecules, including growth factors such as vascular endothelial growth factor^7^, anti-fibrotic factors such as hepatocyte growth factor and prostaglandin E2^8^, angiogenic factors^9^ and molecules that inhibit apoptosis and activate tissue specific progenitor cells. MSC-conditioned medium recapitulates the activity of MSC *in vitro* indicating a paracrine effect that initiates cellular signalling that ultimately enhance tissue repair^10^,^11^. These MSC properties have led to their use in numerous clinical trials for a variety of diseases, including graft versus host disease^12^, cardio-vascular disease as a cell-based therapy^13^ or in tissue-engineered constructs for bone (www.clinicaltrials.gov).

MSC have recently been identified in the highly regenerative uterine lining (endometrium). Human endometrial mesenchymal stem/stromal cells (eMSC), like other mesenchymal stem/stromal cells are a rare group of quiescence cells (~1–4%) found in a perivascular location^14^,^15^. In the endometrium, eMSC are found in the functionalis layer that is shed during menstruation and in the remaining basalis layer from which the new functionalis grows each month^16^,^17^. eMSC can be prospectively isolated from endometrial biopsy tissues using co-expression of the MSC markers, CD140b and CD146 by flow cytometry sorting or with a single marker SUSD2 using magnetic beads^14^,^15^. eMSC isolated using the W5C5 antibody that recognises the SUSD2 antigen have typical *in vitro* MSC properties, in addition to reconstituting stromal tissue *in vivo* and significantly reducing inflammation and promoting neovascularisation when delivered as a tissue-engineering construct in an animal model of wound repair^14^,^18^. SUSD2 is a novel marker, recently identified, as an alternate to CD271 for purifying human bone marrow MSC (bmMSC)^19^. SUSD2 is a type I transmembrane protein that has a large extracellular region with domains known to have roles in cell adhesion, homodimerisation, signal transduction and migration^20^ through interaction with LGALS1 (galactosidase-binding, soluble, 1) and UGGT1 (UDP-glucose ceramide glucosyltransferase-like 1) proteins^21^. SUSD2 is also highly expressed in brain especially in the hippocampus where it plays a role in neuritic growth and excitatory synapses which involve its cell adhesive properties^21^.

eMSC require expansion for use in clinical applications similar to bmMSC^14^,^22^,^23^. However like other MSC, eMSC undergo spontaneous differentiation to fibroblasts during the culture expansion process, decreasing their purity^24^. Heterogeneity and decreased efficacy of culture-expanded MSC result in reduced clinical effect. In addition, the regenerative potential of MSC declines with age^25^. Freshly isolated, culture expanded SUSD2^+^ eMSC underwent spontaneous differentiation indicated by decreasing proportions of SUSD2^+^ cells and increasing SUSD2^−^ cells with increasing passage^18^. The MSC markers designated by the International Society of Cellular Therapy (ISCT) do not indicate the “stemness” of culture expanded MSC. During culture expansion, MSC age losing CFU activity, tri-lineage multipotency, telomere length and ability to generate neotissue *in vivo*, despite expressing the standard ISCT MSC markers^24^,^26^,^27^. For example, bmMSC lose differentiation and proliferative capacity even though expressing high levels of CD44, CD271, CD90 and CD105 during extended culture^28^. Thus, these typical ISCT MSC markers cannot be used to monitor the differentiation state of MSC during culture. The novel marker SUSD2 and CD146 may be superior markers to monitor the status of MSC during the culture expansion process^22^.

The loss of clonogenicity, multipotency and onset of senescence upon extensive culture of bmMSC results in increased senescence-associated beta-galactosidase and p16 gene expression, as well as changes in DNA methylation, limiting the utility of MSC as a cell-based therapy^29^. The maintenance of a stem/progenitor cell population during culture expansion requires activity of signalling pathways involved in self-renewal and proliferation while preventing differentiation^30^. Several small molecules targeting signaling pathways involved in maintaining pluripotency or blocking differentiation have been used for pluripotent cell cultures. Inhibition of the GSK3β, MEK and TGF-β signalling pathways have been used in rat and human induced pluripotent stem cells (iPSCs) to prevent spontaneous differentiation and maintain their stemness during prolonged culture^31^. The ROCK inhibitor, Y27632, has been used to prevent dissociation-induced cell death of human embryonic stem cells^32^. The PDGFR-IV tyrosine kinase inhibitor (#521233, Calbiochem) increased expression of pluripotency genes *OCT4* and *NANOG*, and increased MSC potency from multipotent to a pluripotent state^33^. Transforming-growth factor beta receptor (TGF-βΡ), platelet-derived growth factor receptor (PDGF-R) and basic-fibroblast growth factor receptor (bFGFR) pathways have crucial roles in specifying MSC differentiation into osteogenic, myogenic, adipogenic and chondrogenic lineages^34^. Controlling MSC self-renewal and differentiation with small molecule inhibitors or activators of one or more of these key signalling pathways, should generate a homogeneous MSC population during culture expansion.

The use of eMSC for cell-based therapy requires their expansion in culture conditions that supports homogenous growth and maintains self-renewal and multipotency. A83-01 is a potent selective inhibitor of the TGF-βRs ALK4, 5, and 7. A83-01 inhibits SMAD2 phosphorylation^35^,^36^, maintains self-renewal and proliferation of rat and human induced pluripotent stem cells in cultures without feeder layers^35^. The aim of this study was to determine whether A83-01 maintained growth and prevented spontaneous differentiation of eMSC during culture expansion. In this study, we showed that A83-01 promotes proliferation of late passage SUSD2^+^ cells in serum free medium (SFM), an effect mediated by SMAD2/3 signaling. A83-01 also prevented senescence and apoptosis of cultured eMSC, suggesting that TGF-β has a role in regulating SUSD2 expression and eMSC growth, apoptosis and senescence and therefore may have a role in spontaneous MSC differentiation during culture expansion. Small molecules such as A83-01 may provide an approach for the expansion of undifferentiated MSC for use in tissue engineering and cell-based therapy.

## Results

### A83-01 dose dependently promotes eMSC proliferation

**Figure 1:**
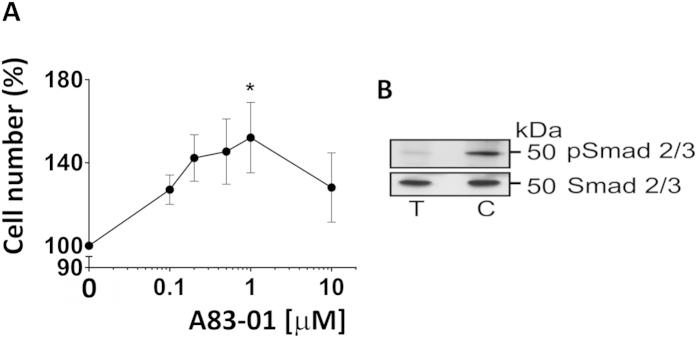
Dose Response curve of A83-01 promotion of eMSC proliferation. **(A)** Passage 3 eMSC incubated A83-01 in SFM in 5%O_2_/5%CO_2_ at 37 °C for 7 days was assessed by MTS cell viability assay. Means for triplicates were obtained for each sample at each concentration, then normalised to vehicle control DMSO (100%) and plotted as mean ± SEM of n = 6 patient samples. **(B)** Passage 6 eMSC lysates with or without 1μM A83-01 were immunoblotted with anti-SMAD 2/3 or anti-pSMAD 2/3 antibodies. A83-01 inhibited TGF-βR-induced phosphorylation of SMAD 2/3. (C = control, T =  treated).

In our earlier studies, we observed that SUSD2^+^ cells diminished in number with increasing passage^18^, despite their high purity on initial seeding following SUSD2 magnetic bead sorting^14^. To examine the effect of the TGF-βR inhibitor, A83-01 on eMSC proliferation, passage 3 eMSC were cultured in SFM in 5% O_2_ with A83-01 concentrations ranging from 0–10 μM for 7 days. Control medium was supplemented with vehicle. The MTS cell viability end-point assay was used to assess the effect of A83-01 on eMSC growth. As shown in Fig. 1A, A83-01 dose dependently increased the number of viable cells with maximal effect at 1 μM concentration (p < 0.05) by day 7. This result suggests that TGF-βR signaling regulates cell growth in negative manner. All further experiments were carried out with P6 eMSC using A83-01 at 1 μM concentration. A83-01 blocks the phosphorylation of SMAD2/3 (Fig. 1B) thus indicating its activity via SMAD pathway.

### Surface Phenotype Expression of A83-01 treated eMSC

**Figure 2:**
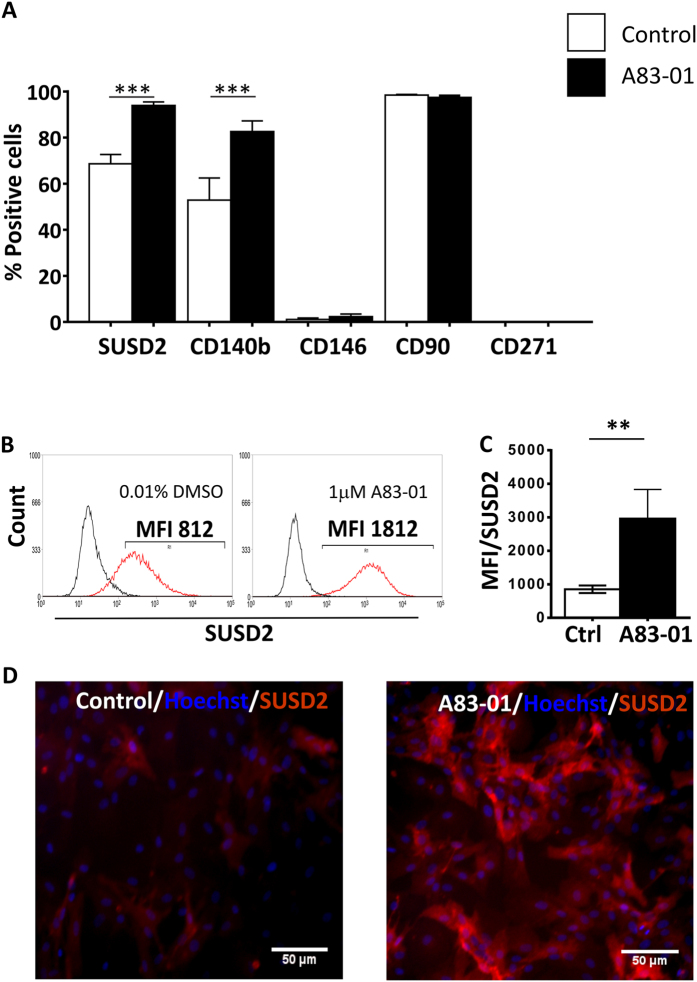
Phenotype of P6 eMSC cultured with or without A83-01 in serum free medium in 5%O_2_. **(A)** % Positive cells for MSC surface markers on passage 6 eMSC (n = 8) cultured in 1μM A83-01(black bars) or in 0.01% DMSO (white bars) for 7 days and assessed by single-colour flow cytometry. **(B)** Representative flow cytometric histograms of SUSD2^+^ eMSC treated with (black bar) and without (white bar) 1 μM A83-01 and MFI summarised in **(C)**. **(D)** SUSD2 expression on control (left panel) and A83-01 treated (right panel) eMSC by immunofluorescence (red). Data are mean ± SEM of n = 8 different patient samples. **p < 0.01, ***p < 0.001.

We next examined the phenotype of A83-01 treated eMSC. Single-color flow cytometry analysis of 5 MSC markers showed that untreated P6 eMSC cultures comprised 69%-SUSD2^+^, 53%-CD140b^+^, 1%-CD146^+^, 95%-CD90^+^ and 0%-CD271^+^ positive cells (Fig. 2A), suggesting loss of the MSC phenotype and spontaneous differentiation. It is noteworthy that CD90, the representative ISCT MSC marker^4^ did not change over the period of culture, and that P6 eMSC did not express CD271 (bmMSC marker) whether incubated with or without A83-01. There was a significant increase in the percentage of SUSD2^+^ (94%, p < 0.05) and CD140b^+^ (83%, p < 0.05) cells when the P6 cells were treated with 1 μM A83-01 for at least 7 days. There was also an increase in the mean fluorescence intensity for the SUSD2 marker on the SUSD2^+^ cells (Fig. 2B,C), indicating an increase in the number of SUSD2 molecules per cell (p < 0.05). This was also evident by immunofluorescence (Fig. 2D). However, A83-01 had no effect on the CD146 expression, which was downregulated during culture expansion (Fig. 2A).

### A83-01 maintained functional properties of late passage eMSC

**Figure 3:**
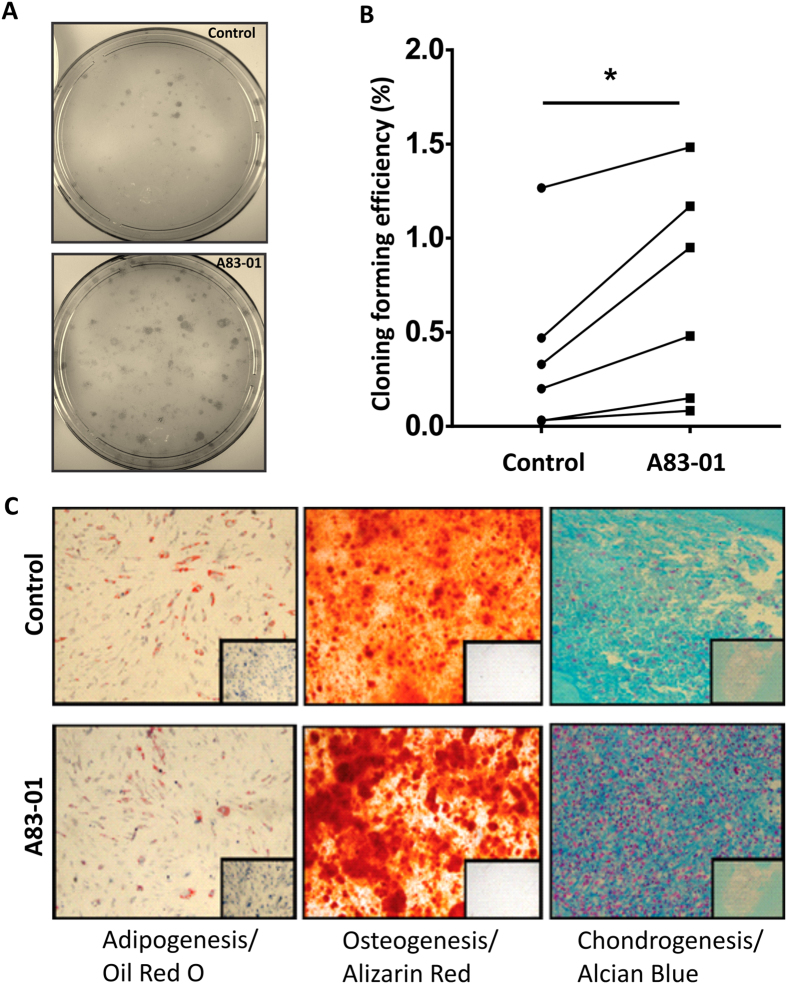
Functional MSC properties of P6 eMSC cultured with or without A83-01 in serum free medium. (**A)** Representative culture plates seeded at clonal density (50 cell/cm^2^). **(B)** Graph shows Colony Forming Efficiency of P6 eMSC pre-treated with 1μM A83-01 or 0.01% DMSO vehicle for 7 days in 5% O_2_ in SFM followed by clonal culture at 50 cells/cm^2^ in SFM for 4 weeks. **(C)** Multilineage mesodermal differentiation of 0.01% DMSO treated control and 1 μM A83-01 treated P6 eMSC showing adipogenic, osteogenic, and chondrogenic differentiation (controls were cultured in 1% serum media) for four weeks in 5%O_2_. Oil Red O was used to visualise cellular lipid vesicles for adipogenic differentiation, Alizarin Red to detect calcium mineralisation for osteogenic differentiation and Alcian Blue to detect acidic polysaccharides in the extracellular matrix for cartilage differentiation. Representative images from n = 3 samples. Data are mean ± SEM of n = 6 different samples *p < 0.05.

We next investigated whether inhibition of the TGF-βR signalling pathway in late passage eMSC cultures altered their MSC functional properties. The cloning efficiency of P6 A83-01 pre-treated eMSC was significantly greater (p < 0.05) than control cells (Fig. 3A,B). The A83-01 pre-treated eMSC also generated larger colonies than untreated eMSC.

Next, we tested whether A83-01 pre-treated eMSC retained MSC multilineage differentiation capacity. P6 eMSC pre-treated with or without 1 μM A83-01 were cultured in differentiation induction media or 1% FCS growth medium (control) to assess differentiation into adipocytes, osteocytes and chondrocytes (Fig. 3C). A83-01 pre-treated and untreated cells showed similar phenotype changes in adiopogenic medium with similar numbers of cells containing Oil Red O stained lipid droplets. Similarly for osteogenic differentiation, the amount of Alizarin Red stained calcium deposits was comparable. In contrast, chondrogenic differentiation of the cell pellets was greater for the A83-01 pre-treated cells, as a strong Alcian Blue stained matrix in a cartilage-like organoid was observed, while the untreated eMSC pellet disintegrated easily with little evidence of chondroitin sulphate matrix deposition (Fig. 3C). There was no differentiation in non-induction medium.

### A83-01 effect on pluripotency and stem cell gene expression

**Figure 4:**
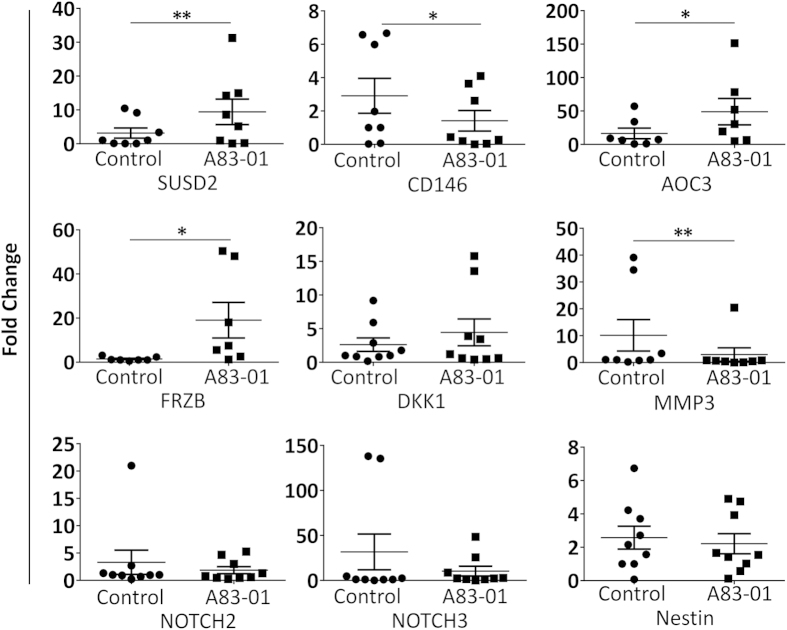
Quantitative RT-PCR analysis of MSC genes. P6 eMSC cultures treated (black squares) or untreated control (black circles) with 1 μM A83-01 in 5%O_2_/5%CO_2_/90%N at 37 °C for 7 days. qRT-PCR analysis of *SUSD2*, *CD146, AOC3, FRZB*, *MMP3, DKK1, NOTCH3, NOTCH2* and *NESTIN*. β-Actin or GAPDH were used to normalise the mRNA level, and fold change was calculated using 2^−ΔΔCT^. Data are mean ± SEM on n = 7 different tissue samples. *p < 0.05; **p < 0.01.

We next examined the expression of pluripotency and MSC genes suggested to have a role in maintaining MSC self-renewal. Quantitative RT-PCR of A83-01 treated and untreated cells failed to detect pluripotency genes *OCT4*, *SOX2* and *NANOG* in either group (results not shown) although they were demonstrated in the human iPS cells positive control. Consistent with the flow cytometry data, *SUSD2* was downregulated in the control group and highly expressed in the A83-01 treated cells (p = 0.0078) (Fig. 4). The expression of *CD146* and *MMP3* genes was reduced in A83-01 treated cells (p = 0.04 and p = 0.0078, respectively) (Fig. 4). There was also an increase in the expression of *AOC3* (p = 0.031), a marker of SUSD2^+^ cells^37^ and *FRZB* (p = 0.015) (Fig. 4), a gene encoding for a Wnt ligand binding protein, in A83-01 treated group while no difference was observed in the expression of *NOTCH2, NOTCH3* and *DKK* genes (Fig. 4).

### A83-01 blocks apoptosis and senescence in P6 eMSC

**Figure 5:**
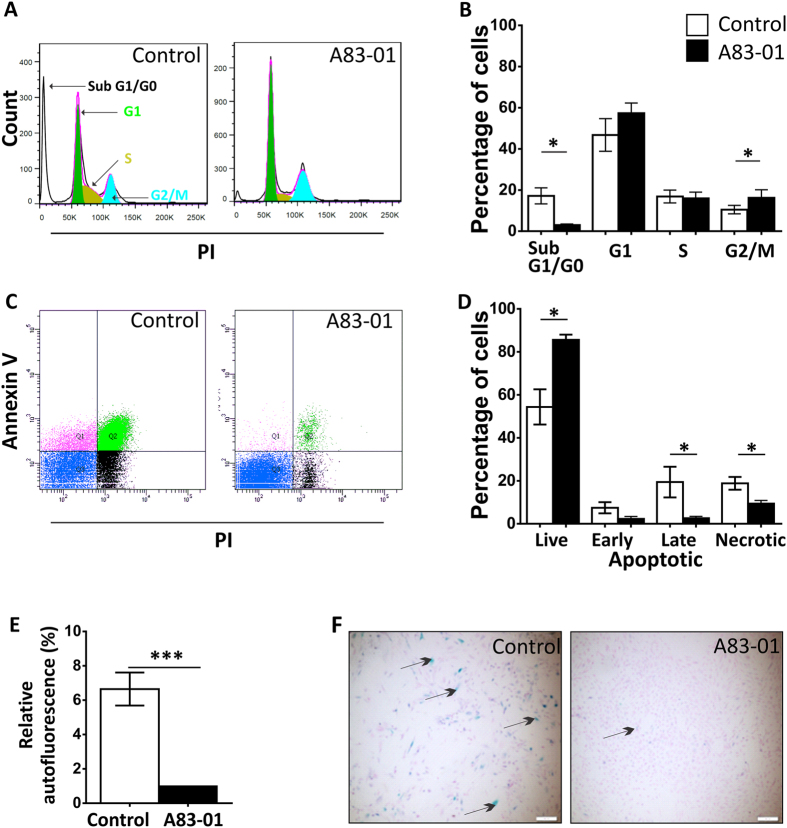
A83-01 blocks apoptosis and promotes eMSC proliferation. P6 eMSC treated with 1 μM A83-01 or 0.01% DMSO cultured for 7 days in SFM in 5%O_2_/5%CO_2_/90%N were assessed by (A) Cell cycle analysis of PI stained cells. Shows representative PI staining on a linear axis of a flow cytometry plot **(B)** the percentage of cells in SubG1/G0, G1, S and G2/M stages of the cell cycle (black bar A83-01 treated, White bar control). Data are mean ± SEM, n = 7 patient samples; *p < 0.05. (C) Annexin-V and PI staining. Representative flow cytometric plots. The lower left quadrant of each panel shows the viable cells, upper left early apoptotic; upper right late apoptotic and lower right necrotic cells and **(D)** graph showing the percentage of live, apoptotic and necrotic cells. Data are mean ± SEM, n = 6 patient samples, *p < 0.05. **(E)** Relative autofluorescence by flow cytometry of unstained P6 cells treated (black bar) and untreated (white bar) with 1 μM A83-01. Data are mean ± SEM, n = 10, ***p < 0.005. **(F)** Representative images showing the staining of senescence-associated β-galactosidase (SA-β-gal) in cultured P6 eMSC treated with or without 1 μM A83-01.

To identify the mechanism of action of A83-01 in increasing eMSC proliferation (Fig. 1), we undertook cell cycle analysis (Fig. 5A) with propidium iodide to label DNA. Figure 5A,B shows that A83-01 treatment increased the proportion of cells in G2/M phase (p < 0.05) indicative of an increased rate of cell division. There were also significantly fewer A83-01 treated cells in the sub G1/G0 phase of the cycle compared with control cells indicating fewer apoptotic cells with fragmented DNA content in the A83-01 treated cells (Fig. 5A,B). We then quantified the apoptotic cells using Annexin V flow cytometry to assess early phase apoptosis. The inclusion of PI was to detect late apoptotic and necrotic cells. Culture expanded P6 eMSC significantly reduced the percentage of live cells and increased the proportion of apoptotic cells as shown by the increased binding of Annexin V to exposed phosphatidylserine (PS) on the outer leaflet of the plasma membrane (Fig. 5C,D). The increased PI staining in the untreated eMSC also indicated increased necrotic cells. These changes were mitigated by pre-treatment with 1 μM A83-01 (p < 0.05) (Fig. 5C,D).

To further understand the action of A83-01, we examined unstained P6 eMSC from treated and control groups by UV light to quantify autofluorescence, as a measure of senescence. As shown in Fig. 5E, control P6 eMSC were significantly more autofluorescent than the A83-01 treated P6 eMSC (p = 0.001). Therefore, we measured senile associated β-Gal (SAβ-Gal) activity by incubating cells with X-Gal. As shown in Fig. 5F, A83-01 treated P6 eMSC showed little β-Gal staining whereas the untreated control eMSC displayed blue staining indicative of senescent cells. Furthermore the A83-01-treated cells were smaller and more numerous, in agreement with our findings above.

## Discussion

The main findings from this study are that A83-01, a small molecule TGF-βR inhibitor, prevented the typical loss of undifferentiated MSC during culture expansion. Specifically, we showed that A83-01 treatment prevented loss of SUSD2^+^ eMSC in late passage cultures by promoting the mitosis and proliferation of P6 SUSD2^+^eMSC and by preventing their apoptosis and senescence. A83-01 treated SUSD2^+^ cells in late passage culture retained their MSC properties, showing greater clonogenicity then untreated cells. In particular there were greater numbers of large colonies which undergo serial cloning and are more proliferative than those initiating small colonies^38^. Their multilineage differentiation capacity was maintained as well as expression of key MSC genes; *SUSD2*, *AOC*3^37^ and *FRZB*^39^. We further identified that the signalling pathway blocked by A83-01 was TGF-βR mediated apoptosis via SMAD2/3 phosphorylation. Since multiple pathways work together in regulating MSC fate and TGF-βR pathway signalling is pleiotropic, targeting this pathway may provide an ideal method for maintaining undifferentiated MSC in cell production protocols for clinical use.

We showed culture expansion of eMSC lead to a loss of clonogenicity and expression of SUSD2, CD140b and CD146 surface markers while CD90, a standard ISCT MSC marker does not change. This was also shown by loss of SUSD2^+^-expressing eMSC when induced by TGF-β1 to differentiate into smooth muscle cells^40^. These properties support the concept that eMSC spontaneously differentiate into fibroblasts lacking the expression of perivascular markers, clonogenicity, and osteogenic and chondrogenic differentiation capacity. Further molecular characterisation of differentiation at the transcript and protein levels is feasible with qRT-PCR and western blotting respectively. TGF-βR signalling is necessary for chondrogenic differentiation^34^. However, while the experimental medium for eMSC culture expansion contained A83-01, the chondrogenic differentiation medium contained TGF-β1 without A83-01 to assess chondrogenic differentiation potential of A83-01 treated and untreated cells. One advantage of using small molecules rather than siRNA to modulate receptor activity is that their inhibitory effect is reversed as soon as the small molecules are removed. We were therefore able to show that chondrogenic differentiation was enhanced in A83-01 pre-treated cells. A83-01 not only increased the expression of SUSD2 proteins but also CD140b. In contrast, CD146 gene expression was greater in the untreated group but did not appear to be translated into protein as it was not detected by flow cytometry. Furthermore, expression of CD146 on cultured MSC is regulated by factors such as hypoxia, growth factors, and metalloproteases^41^,^42^. Culture expanded MSC are more autofluorescent than the primary cells indicating replicative senescence and loss of proliferative ability^43^,^44^ an effect observed in P6 control eMSC which was mitigated by A83-01 treatment.

Endometrial MSC are an attractive source of cells for tissue engineering and cell-based therapies because they can be harvested with minimal discomfort to patients, have standard MSC properties *in vitro* and *in vivo*, and they can be cultured in serum free conditions, offering a readily available cell source for allogeneic as well as autologous use. The necessity to expand MSC for clinical use due to their rarity and their subsequent spontaneous differentiation limits the full potential of eMSC and MSC in general.

TGF-β belongs to a superfamily of TGF cytokines which has multiple functions. TGF-β plays a vital role in MSC differentiation along with PDGF and FGF-2 pathways^34^,^45^. TGF-β is synthesized by endometrial stromal cells under the influence of physiological female hormones, fluctuating during different phases of menstrual cycle. TGF-β production increased in the secretory and menstrual phases but was diminished in the proliferative phase, suggesting that TGF-β promotes differentiation^46^. Similarly, TGF-β promoted differentiation of SUSD2^+^ eMSC when cultured on polyamide/gelatin meshes^40^. TGF-β can independently, as well as in association with Wnt and NOTCH signaling pathways, regulates proliferation and differentiation of MSC^47^,^48^. Gene profiling of purified eMSC shows that there is an increased fold change in FRZB receptor indicating an interaction with the Wnt signalling pathway. Pluripotency genes are not present in freshly isolated eMSC^39^ and we found that P6 eMSC did not express pluripotency markers nor did A83-01 treatment upregulate their expression.

Apoptosis or programmed cell death mainly results from activation of cellular caspases. The TGF-βR pathway also participates in apoptosis via SMAD activation, in association with Death associated protein 6 and TGF-βR inducible transcription factor^49^,^50^. In epithelial cells and hepatocytes, TGF-βR induces apoptosis and inhibits proliferation^49^,^50^,^51^ through the activation of TGFβ-inducible early gene-1 (TIEG1) via phosphorylation of SMAD2/3 and formation of reactive oxygen species. TIEG1 inhibits cell growth and leads to apoptosis^49^. P6 eMSC treated with 1 μM A83-01 showed significantly increased growth by preventing apoptosis compared to the untreated cells, however it is not known if TIEG1 is involved.

Human Lgr5^+^ liver stem cell cultures in mouse culture medium was not supported for more than three weeks and revealed highly active TGF-β signalling. This was also mitigated by blocking with A83-01 which improved cloning efficiency with extended time in culture of the liver stem cells^52^. A83-01 is a selective inhibitor of TGF-βR type I ALK4/5/7. It has a thiourea group in its structure, conferring copper ion chelation properties. Thioureas prevent copper mediated oxidative cellular damage by chelating copper in the medium^53^. One of the potential mechanisms by which A83-01 acts in SUSD2^+^ eMSC may be the prevention of spontaneous differentiation and apoptosis mediated via the copper chelating thiourea moiety. Our study also demonstrated for the first time that TGF-βR signalling is an essential negative regulator of SUSD2 expression through SMAD2/3 signalling in eMSC. In addition, the TGF-βR pathway is involved in regulating eMSC proliferation, senescence and apoptosis.

## Conclusions

In summary we have shown that TGF-βR signaling is involved in eMSC cell fate *in vitro*. A83-01, a small molecule TGF-βR inhibitor, enhanced the expression of SUSD2 and CD140b, maintaining eMSC clonogenic phenotype during prolonged culturing, promoting cell proliferation and preventing apoptosis and senescence. Small molecules such as A83-01 that promote eMSC proliferation in the undifferentiated state may provide an approach for the expansion of undifferentiated MSC for use in tissue engineering and cell-based therapies.

## Materials and Methods

### Human endometrial tissue samples

The experimental protocols were conducted under the ethical guidelines according to the National Health and Medical Research Council (NHMRC) of Australia’s National Statement on Ethical Conduct in Human Research. Human ethics approval was obtained from the Monash Health and Monash University Human Research Ethics committees. Human endometrial tissues samples were collected from pre-menopausal women who were undergoing endometrial curette or hysterectomy for non-endometrial pathologies and who were not taking any exogenous hormones for three months prior to the surgery, following informed patient consent.

### Isolation and magnetic bead sorting of SUSD2^+^ eMSC

eMSC were isolated according to our previously published protocol^14^. Briefly, endometrial tissues from hysterectomy sample were carefully scraped off the underlying myometrium. Both hysterectomy and curette tissues were mechanically minced and digested with 0.5% collagenase type I and 40 μg/ml deoxyribonuclease type I (both Worthington Biochemical Corporation) in Dulbecco’s modified Eagle’s medium (DMEM/F12) for 90 and 60 minutes, respectively in a humidified incubator at 37 °C on a rotating MACSmix (Miltenyi Biotech). The tissue digest was filtered through 40 μm cell strainer (BD Biosciences) to separate the epithelial gland fragments and undigested tissues. The red blood cells in the filtrate were separated from the single stromal cells by density gradient centrifugation using Ficoll-Paque (GE healthcare Bio-science). eMSC were obtained by incubating the stromal cells in Phycoerythrin (PE)-conjugated anti-human SUSD2 (10 μg/ml, BioLegend)) in 0.5% FCS/PBS (bead medium) and anti-PE magnetic-activated cell sorting microbeads (Miltenyi Biotec) for 30 minutes each in the dark on ice. The conjugated pellet was resuspended in bead medium and applied to a Miltenyi column (Miltenyi Biotec, #130-042-201) in a magnetic field. The separated cells, containing the SUSD2^+^ eMSCs in the column were eluted in bead medium and the cells counted.

### Cell culture and assessment of cell proliferation

The SUSD2^+^ eMSC were initially maintained in DMEM/F12 medium containing 10% Fetal calf serum (FCS) (Invitrogen), 1% antibiotic-antimycotic (Life Technologies) and 2 mM glutamine and slowly changed to an in-house DMEM/F12 serum free medium supplemented with basic fibroblast growth factor (FGF2, 10ng/ml) and epidermal growth factor (EGF, 10 ng/ml) (SFM) at 37 °C in 5%O_2_/5%CO_2_/90%N, as described previously^22^. The cells were seeded at 5000 cells/cm^2^ density at subsequent passages in fibronectin (10 ug/ml) pre-coated culture flasks. Cell proliferation assays were performed at passage 3 by seeding 1000 cells in 100 μl SFM per well in fibronectin pre-coated 96-well plates with or without A83-01, concentrations varying from 0–10 μM. Media was changed every second day and contained A83-01 at the same concentration. Following 7 days of culture, 20 μl of MTS tetrazolium reagent (Promega) was added to each well and incubated for 2 hours and the soluble formazan product was quantified using a micro plate reader (SpectraMax Plus384; Molecular Devices) at 490 nm. Further experiments were done at passage 6 where the cells were separated into two groups, one group was treated with 1μM A83-01 and the control with (0.01% DMSO) vehicle. The data was normalised to the control and reported as a percentage.

### Immunophenotyping

eMSC were trypsinised with TrypLE^TM^ (Life technologies, #12604-021)) and resuspended at 10^5^ cells/tube. Cells were washed with 5% heat-inactived newborn calf serum in DMEM (bench medium) and incubated with PE-, APC- or FITC-conjugated primary antibodies or matched-isotype controls in bench medium for 30 minutes in the dark on ice. Primary antibody used was CD146 (1:1 supernatant, clone CC9, kind gift from Prof David Haylock CSIRO). PE-conjugated antibodies were SUSD2 (1:20, Biolegend, #327406), CD140b (1:20, R&D systems FAB1263P) and CD271 (1:20, Miltenyi Biotec). APC-conjugated antibody was CD90 (1:20, BD Pharmingen). Isotype control antibodies at the same concentration as the primary antibody were included for each run and were used to set the electronic negative control gate on the flow cytometer. Finally, cells were washed with bench medium and fixed with 4% paraformaldehyde (PFA) in 2%FBS/PBS. Samples were analysed using a MoFlo Flow Cytometry (Beckman Coulter) and Summit software (version 5.2., Beckman Coulter).

### Immunofluorescence microscopy

Passage 6 (P6) eMSC were cultured on coverslips with or without 1 μM A83-01 for 7days and then fixed in 4% PFA followed by protein block (Dako, X0909) for 10 minutes each at room temperature with washing in between with PBS. PE-conjugated SUSD2 antibody (1:200, BioLegend, #327406) in 2% FCS/PBS was incubated for 2 hours at room temperature in dark. Isotype control IgG1 antibody was used as a negative control. Hoechst 33258 (1:2000, Molecular Probes) was used to visualise nuclei. Images were visualised and photographed using a Delta Vision microscope, and analysed using ImageJ software (ImageJ-win32.Ink).

### Immunoblotting

Cell lysates were prepared using lysis buffer (50 mM Tris pH 8.0, 150 mM NaCl, 1% triton X-100) with mini protease inhibitor cocktail tablet (Roche) and phosphatase inhibitor sodium vanadate (2 mM). The following antibodies were used: anti-SMAD 2/3 antibody (#3102S), antiphospho-SMAD 2/3 (#8828S), horseradish peroxidise conjugated secondary antibody (#7074S) from Cell Signalling Technology. The specific protein was detected by treating the membrane for two minutes with enhanced chemiluminescence (# 133406, Abcam) which provides the HRP substrate, and capturing the signal in X-ray films.

### Quantitative RT-PCR

RNA was isolated using PureLink^®^ RNA mini Kit (Life technologies, #12183018A) and further treated with DNase (PureLink^TM^ DNase, Invitrogen) to obtain DNA-free total RNA. First-strand cDNA was synthesized using SuperScript III first-strand synthesis system (Invitrogen). 50 ng of cDNA was amplified and detected using TaqMan probes for *OCT4, NANOG* and *SOX2*, and SYBR Green Super Mix for *SUSD2, CD146, AOC3, MMP3, FRZB, DKK1, NOTCH3, NOTCH2* and *NESTIN.* The PCR conditions consisted of initial denaturation at 95 °C for 10 minutes, followed by 40 cycles of denaturation at 95 °C for 15 seconds and annealing/polymerisation at 60 °C for 60 seconds. Primer sets are detained in Table 1. GAPDH or β-Actin was used as an endogenous control to normalise the target gene expression and fold change was calculated using the 2^−ΔΔCT^ method.

### Mesenchymal stem/stromal cell properties

To assess colony-forming ability, P6eMSC pre-treated and untreated with 1 μM A83-01 for 7 days were seeded at 50 and 100 cells/cm^2^ on fibronectin-coated 100 mm culture dishes (BD Falcon) in SFM in a tri-gas incubator 5%O_2_/5%CO_2_/90%N for four weeks. The cells were then formalin-fixed for 10 minutes and stained with haematoxylin (AMBER SCIENTIFIC). The colonies were washed twice with distilled water and counterstained with Scott’s tap water to develop the blue colour. Colony efficiency was calculated by counting the number of colonies divided by the number of cells seeded and the percentage determined.

To assess multipotency, the remaining cells were cultured in adiopogenic and osteogenic, and control medium (1% fetal calf serum) on 13-mm coverslips, and for chondrogenic differentiation the cells were cultured as 3D pellets in chondrogenic induction media for 4 weeks at 37 °C in 5%CO_2_/5%O2 as described previously^22^. To detect the differentiation, the cells were fixed with 4% PFA and incubated with 1% Oil Red O for adipogenesis, 4% Alizarin Red (pH 4.1) for osteogenesis and 1% Alcian blue (pH 2.5) on paraffin embedded sections (5 μm) of the micromass pellet for chondrogenesis. Stained cells were examined under an Olympus BX41 microscope (Olympus) and images were taken with 10X objective lens using the DP25 digital camera (Olympus).

### Cell cycle analysis and apoptosis by flow cytometry

To assess the cell cycle status, P6 A83-01 treated and untreated cells were detached, pelleted and fixed in ice-cold 70% ethanol at 4 °C overnight. They were washed with 2%FBS/PBS and incubated with 50 μl RNAse (100 μg/ml, Sigma) at room temperature for 15 minutes. 200 μl of propidium iodide (PI) (50 μg/ml, Sigma P4170) was added and the cells were analysed immediately by flow cytometry using BD FACS Canto^TM^ II on PI-linear scales. The data were analysed using Flow Jo 7.6.3.

To assess apoptosis, P6 A83-01 treated and untreated cell-pellets were stained with Annexin V-APC/PI kit following the manufacturer’s protocol (#88–8007, eBioscience). Briefly, cells were trypsinised and resuspended in 100 μl binding buffer, 5 μl of Annexin V-APC solution was added to the cell suspension and incubated for 15 minutes at room temperature protected from light. Following washing with the binding buffer, 5 μl of PI was added to the cells suspended in 200 μl binding buffer and events immediately acquired by flow cytometry using BD FACS Canto^TM^ II and analysed with Flow Jo 7.6.3.

### Cell senescence by β-galactosidase and auto-fluorescence

Senescent cells were assessed by staining for beta-galactosidase activity. P6 A83-01 treated and untreated eMSC were cultured on coverslips for 7 days as described above, then fixed in 4% PFA for 10 minutes and stained in freshly prepared X-Gal (1 mg/ml in DMSO) staining reagent (5 mM K_3_Fe(CN), 5 mM K_4_Fe(CN), 2 mM MgCl_2,_ 150 mM NaCl) in citrate buffer at pH6 for 24 hours at 37 °C. The cells were washed twice with PBS and counter stained with nuclear fast red (Sigma-Aldrich, 0.1% w/v) for 10 minutes, then examined under an Olympus BX41 microscope (Olympus). Images were taken with 10X objective lens using the DP25 digital camera (Olympus).

### Statistical Analysis

Non parametric Friedman’s test with Dunn’s multiple comparison post hoc tests were used to test for multiple groups and Wilcoxon matched-pairs signed rank tests were used to test for statistical significance between treated and control groups. Data are presented as mean ± standard error of mean. Differences were considered statistically significant at p < 0.05.

## Additional Information

**How to cite this article**: Gurung, S. *et al.* Inhibition of Transforming Growth Factor-β Receptor signaling promotes culture expansion of undifferentiated human Endometrial Mesenchymal Stem/stromal Cells. *Sci. Rep.* **5**, 15042; doi: 10.1038/srep15042 (2015).

## Figures and Tables

**Table 1:** RT-PCR Primers Used.

GENE	PRIMER SEQUENCE
OCT4	F:CAGTGCCCGAAACCCACAC
	R: GGAGACCCAGCAGCCTCAAA
NANOG	F: TAATAACCTTGGCTGCCGTCTCTG
	R: GCCTCCCAATCCCAAACAATACGA
SOX2	F: ACACCAATCCCATCCACACT
	R: GCAAACTTCCTGCAAAGCTC
SUSD2	F: AGAGCTGGATGGACCTGAAA
	R: ATGCCAGCATGATGGAGAC
CD146	F:GAAGCATGGGGCTTCCCAG
	R:CCTCCGGAGCTTTGTAGACG
AOC3	F:TCAGCTGGGAGAGGATTTGG
	R:CGGAAGTAGATGGAGTCGGC
MMP3	F:AGCAAGGACCTCGTT TTCATT
	R:GTCAATCCCTGGAAAGTCTTCA
FRZB	F:CCTGCCCTGGAACATGACTAA
	R:CAGACCTTCGAACTGCTCGAT
NESTIN	F:GAAACAGCCATAGAGGGCAAA
	R:TGGTTTTCCAGAGTCTTCAGTGA
DKK1	F:GATCATAGCACCTTGGATGGG
	R:GGCACAGTCTGATGACCGG
NOTCH2	F: GTTTGTGTGGATGGGGTCAA
	R: TCCACATCCTCTGTGCAGAA
NOTCH3	F:GGACCTGCCGTGGCTATA
	R:ACGTCGTCCTCACAGTTATCA
GAPDH	F:TGTGGGCATCAATGGATTTGG
	R:ACACCATGTATTCCGGGTCAAT
β-ACTIN	F:GGGCATGGGTCAGAAGGATT
	R:AGTTGGTGACGATGCCGTG
